# Validation of the functions and prognostic values of synapse-associated proteins in lower-grade glioma

**DOI:** 10.1042/BSR20210391

**Published:** 2021-05-27

**Authors:** Han Lin, Yong Yang, Chongxian Hou, Yuqing Huang, Liting Zhou, Jiantao Zheng, Guangzhao Lv, Rui Mao, Shanwei Chen, Peihong Xu, Yujun Zhou, Peng Wang, Dong Zhou

**Affiliations:** 1 Department of Neurosurgery, Guangdong Provincial People’s Hospital, Guangdong Academy of Medical Sciences, Guangzhou, China; 2 Shantou University Medical College, Shantou, China; 3 School of Biology and Biological Engineering, South China University of Technology, Guangzhou, China; 4 International Department, Affiliated High School of South China Normal University, Guangzhou, China; 5 School of Medicine, South China University of Technology, Guangzhou, China; 6 Southern Medical University, Guangzhou, China

**Keywords:** lower-grade glioma, seizure, Synapse, synapse associated protein

## Abstract

Synapse and synapse-associated proteins (SAPs) play critical roles in various neurodegeneration diseases and brain tumors. However, in lower-grade gliomas (LGG), SAPs have not been explored systematically. Herein, we are going to explore SAPs expression profile and its clinicopathological significance in LGG which can offer new insights to glioma therapy. In the present study, we integrate a list of SAPs that covered 231 proteins with synaptogenesis activity and post synapse formation. The LGG RNA-seq data were downloaded from GEO, TCGA and CGGA database. The prognosis associated SAPs in key modules of PPI (protein–protein interaction networks) was regarded as hub SAPs. Western blot, quantitative reverse transcription PCR (qRT-PCR) and immunochemistry results from HPA database were used to verify the expression of hub SAPs. There were 68 up-regulated SAPs and 44 down-regulated SAPs in LGG tissue compared with normal brain tissue. Data from function enrichment analysis revealed functions of differentially expressed SAPs in synapse organization and glutamatergic receptor pathway in LGGs. Survival analysis revealed that four SAPs, GRIK2, GABRD, GRID2 and ARC were correlate with the prognosis of LGG patients. Interestingly, we found that GABRD were up-regulated in LGG patients with seizures, indicating that SAPs may link to the pathogenesis of seizures in glioma patients. The four-SAPs signature was revealed as an independent prognostic factor in gliomas. Our study presented a novel strategy to assess the prognostic risks of LGGs, based on the expression of SAPs.

## Introduction

Glioma is the most prevalent neuroepithelial malignant tumor associated with poor prognosis, high disability and high recurrence rates. Depending on the degree of malignancy, gliomas are classified into four grades (WHO grades I, II, III and IV) [1]. Among these, diffuse low-grade glioma (grade II glioma) and intermediated-grade glioma (grade III glioma) make up lower-grade gliomas (LGG) for the similar prognostic effect of isocitrate dehydrogenase (IDH) status in both tumor grades [1,2]. The variability in the prognosis and survival of LGG is attributed to the heterogeneity of its clinical behavior [3]. LGG patients usually manifest as seizures, but in the course of the disease, glioma can also cause neurological and neurocognitive disorders or premature death. The impact of LGG surgical resection depends on the molecular subtype of the tumor and increases with the degree of tumor malignancy [4]. Therefore, more investigations on the LGG and their biomarkers are essential for advanced therapy.

Classified as chemical synapse and electrical synapse, synapse is specialized cytosolic structure in the nervous system. Electrical synapse comprises gap junctions, forming the channel to transport ion and producing electrical flux [5]. On the other hand, the chemical synapse consists of pre-synapse, synaptic cleft, post-synapse, which constructs the complex connection between neuron–neuron and neuron–glia, brick the intricacies and the sophistication of neuronal network configuration [6]. Synapse-associated protein (SAP) is the protein associated with synapse structure and formation, including neurotransmitter receptor, vesicle-associated protein, synaptogenesis factors, among others [7–9]. Mutations or dysregulation of SAPs in the central nervous system causes synapse dysfunction, thereby promoting the progression of various neurodegenerative diseases [10–15]. Recent studies reported that SAPs were significantly associated with malignancies in the brain. Breast metastases express neuroligin-2 and PSD-95 to form pseudo-tripartite synapses with neurons, which permits metastases to utilize glutamate and promote invasion in the brain [16]. A study by Varun Venkataramani et al*.* [17] demonstrated that synapses-like structure is formed between presynaptic neurons and postsynaptic glioma cells. Neuronal-to-glioma synapse (NGS) induced excitatory postsynaptic currents through AMAPR, which significantly contributes to the invasion and growth of glioma [17]. In high-grade glioma (glioblastoma and grade III astrocytoma), the excitatory signal from NGS depolarizes glioma cells and amplify the signals via gap junction, thus promoting their proliferation [18]. Furthermore, targeting neuroligin-3 (important synaptogenesis factors) effectively minimizes the growth of glioma [19]. While acknowledging the roles of SAPs in the occurrence and development of high-grade glioma and breast metastasis, their functions in lower-grade glioma development are subtle. Therefore, additional and systematic research on SAPs will comprehensively impart knowledge about their functions in LGGs.

In this work, LGG RNA-seq and corresponding clinicopathological data were downloaded from gene expression omnibus (GEO) database, the cancer genome atlas (TCGA) database and Chinese glioma genome atlas (CGGA). Then, differentially expressed SAPs between tumoral and normal LGG samples were screened, and their potential biofunction explored. Our findings demonstrated that LGG-related SAPs might advance understanding of the glioma progression and manifestation hence providing insights into developing biomarkers for effective diagnosis and prognosis.

## Methods

### Screening of synapse-associated protein (SAP) and data processing

A list of human SAPs from five sources were integrated including, Venkatesh, Shen, Colón, Jüttner R, Gene Ontology (GO) project (Supplementary Table S1) [18,20–22]. Both RNA-sequencing data and clinical data for Rembrandt were downloaded from Gene Expression Omnibus database (GSE68848, GEO, https://www.ncbi.nlm.nih.gov/geo/), which contained 183 lower-grade glioma (LGG) samples and 28 normal brain tissue samples. Data from Henry Ford Hospital were downloaded from GEO (GSE4290, https://www.ncbi.nlm.nih.gov/geo/), which contained 76 LGG samples and 23 normal brain tissue samples. Using the Limma R-package, differentially expressed genes between normal brain tissue and LGG were identified. Furthermore, 523 and 576 LGG samples with corresponding clinical data were downloaded from the Cancer Genome Atlas database (TCGA, https://portal.gdc.cancer.gov/) and Chinese Glioma Genome Atlas (CGGA, http://www.cgga.org.cn/) respectively.

### Functional enrichment analysis of differentially expressed SAPs

The Database for Annotation, Visualization and Integrated Discovery (DAVID, https://david.ncifcrf.gov/) integrates biological data and analysis tools, providing systematic and comprehensive biological function annotation information for large-scale gene or protein list, and helps users understand biological information [23,24]. Through DAVID, Gene Ontology (GO) component and Kyoto encyclopedia of genes and genomes (KEGG) analyses were performed on differentially expressed SAPs to determine their biofunctions (*P*<0.05 and FDR<0.05). WEB-based Gene Set Analysis Toolkit (WebGestalt, http://www.webgestalt.org/) is a functional enrichment analysis web tool designed for genomic, gene expression, proteomic and large-scale genetic studies from which high-throughput datasets are generated, complementing and extending the functionality of similar data mining tools [25]. In the present study, we used WebGestalt to validate the functional enrichment result from DAVID.

### Protein–protein interaction network construction

Using the STRING database (https://string-db.org/), the protein–protein interaction (PPI) between all differentially expressed SAPs was assessed, and their network was constructed using Cytoscape 3.7.1 [26,27]. Disconnected nodes were removed from the network. Then, the MCODE (Molecular Complex Detection) function was applied to screen key modules from the network (score> 7 and node number > 5) [28].

### Prognosis-related hub SAPs selection

The univariate Cox regression model was used to determine the prognostic significance of SAPs in key modules. Subsequently, the least absolute shrinkage and selection operator (LASSO) was exploited to identify candidate SAPs for further analysis (iteration = 1000) [29]. Then, we developed a multivariate Cox proportional hazards regression analysis and screen for hub SAPs from candidate SAPs.

### Mutation and copy number alteration of hub SAPs

Based on 2200 LGGs from TCGA, MSK, MSLCC and UCSF, mutation and copy-number alteration data for all hub SAPs were identified via the GISTIC algorithm and segmentation analysis in cBioPortal6 (http://www.cbioportal.org/) [30,31]. Overall survival (OS) is computed between tumor samples that have alteration in SAPs and tumor samples that do not. The results are displayed as Kaplan–Meier plots with *P* values from a log-rank test.

### Expression and prognostic value verification of hub SAPs

Immunohistochemistry result from the Human Protein Atlas (HPA) online database (Protein Atlas version 20.1, http://www.proteinatlas.org/), quantitative reverse transcription PCR (qRT-PCR) and Western blot were used to detect the expression of hub SAPs at a translational level [32–34]. GRIK2 staining result of brain tissue are available at https://www.proteinatlas.org/ENSG00000164418-GRIK2/tissue/cerebral+cortex; GRIK2 staining result of glioma are available at https://www.proteinatlas.org/ENSG00000164418-GRIK2/pathology/glioma; GRID2 staining result of brain tissue are available at https://www.proteinatlas.org/ENSG00000152208-GRID2/tissue/cerebral+cortex; GRID2 staining result of glioma are available at https://www.proteinatlas.org/ENSG00000152208-GRID2/pathology/glioma; GABRD staining result of brain tissue are available at https://www.proteinatlas.org/ENSG00000187730-GABRD/tissue/cerebral+cortex; GABRD staining result of glioma are available at https://www.proteinatlas.org/ENSG00000187730-GABRD/pathology/glioma. The survival R-package was used to evaluate the prognostic value of the hub SAPs in LGGs with the Kaplan–Meier technique. Also, the association between the expression of hub SAPs and clinicopathologic features was evaluated.

### Predictive model construction

Based on the hub SAPs, we developed a multivariate Cox proportional hazards regression model to predict the prognosis of LGGs. The risk score of each sample was calculated using the formula:

$$risk\;Score=\sum_{i}^{n}\text{expression level of hub SAP(}i\text{) * }\beta (i)\;(\beta :\text{coefficient of hub SAP})$$

LGGs in the TCGA cohort were subdivided into high-risk groups and low-risk groups according to the median risk score. A log-rank test was performed to compare the overall survival (OS) difference between low-risk and high-risk groups. ROC (receiver operating characteristic) curve was plotted to estimate the performance of the predictive model. Next, LGGs from the CGGA cohort and Rembrandt cohort were used as the validation set to verify the predictive capability of our model. Nomogram is a useful method for predicting the prognosis of cancer patients by simplifying the complex statistical prediction model into a profile chart for assessing the probability of OS in individual patients [35]. In our study, we included all clinical pathological prognostic factors selected from Cox regression analysis to construct a nomogram which can assess the OS probability of 1, 3 and 5 years in LGG patients. The calibration plots were implemented to evaluate the predictive probability of nomogram.

### Quantitative reverse transcription PCR (qRT-PCR)

A total of 15 samples, including 10 samples from patients with LGG and 5 from peritumoral brain region, were used for qRT-PCR verification of the expression of hub SAPs. Briefly, total RNAs were isolated using a Trizol reagent (AG21102, Accurate Biotechnology, Hunan, China). Then, 0.5 μg of the total RNA was used from cDNA synthesis using the PrimeScript RT Master Mix (AG11706, Accurate Biotechnology, Hunan, China). PCR was performed using the SYBR GREEN Kit (AG11701, Accurate Biotechnology, Hunan, China) in the BIO RAD Real‐Time PCR System (Applied Biosystems). GAPDH, the housekeeping, was used as an endogenous control gene. The primers used in this study are listed in Table 1. We duplicated our experiment on every samples for each gene and analyzed the result of Ct. The mRNA expression was quantified as 2^−ΔΔCt^ and presented as means with SD [36].

**Table 1:** List of primers for hub SAPs

Gene	Forward	Reverse
ARC	5′-GTTCATCGTTCTGCCTTGTC-3′	5′-CAGCCTTGAGGATTGGTTATG-3′
GABRD	5′-AGAGCTACGGTTACTCATCGG-3′	5′- GGCCAGCGGACTTGAAGTT -3′
GRID2	5′-GCAACAGGAATGATGACTACACT-3′	5′-CAGGCATACTCTGTGACCACT-3′
GRIK2	5′- TGATGTTGAGCCCTACCGATA-3′	5′-GTTCCATCGACCACTTTTCAATG-3′
GAPDH	5′-GCCATCACAGCAACACAGAA-3′	5′- GCCATACCAGTAAGCTTGCC -3′

Abbreviations: ARC, activity-regulated cytoskeleton associated protein; GABRD, gamma-aminobutyric acid type A receptor subunit delta; GAPDH, glyceraldehyde-3-phosphate dehydrogenase; GRID2, glutamate ionotropic receptor delta type subunit 2; GRIK2, glutamate ionotropic receptor kainate type subunit 2; SAP, synapse-associated protein.

### Western blot analysis

The total proteins (40 μg) were extracted from three pairs of tumor tissue and their corresponding peritumoral tissue using RIPA lysis buffer (Beyotime, Shanghai, China) for 15 min. Then, the proteins were quantified by a commercial BCA Kit (Thermo, U.S.A.), separated by 10% SDS-PAGE and wet-transferred onto polyvinylidene difluoride membranes. The membranes were blocked with 5% skim milk for one hour. Then, we incubated them with primary antibodies (anti-ARC, 1:5000; anti-GRID2, anti-GRIK2, anti-GABRD, 1:1000, Abcam, U.S.A.) overnight at 4°C (Table 2). Next, horseradish peroxidase-conjugated anti-rabbit IgG was used as the secondary antibodies (1:5000, Abcam, U.S.A.) at ambient temperature for 1 h. An anti-GAPDH antibody (1:5000, Proteintech, China) was used to detect the level of GAPDH as an internal control. Protein was visualized using the ECL Plus detection system (GE Healthcare, WI, U.S.A.).

**Table 2:** Antibody information for Western blotting

Name of antibody	Manufacturer	Manufacture code	RRID	Concentration
GAPDH	Proteintech Group	10494-1-AP	AB_2263076	1:5000
GABRD	Proteintech Group	15623-1-AP	AB_2107260	1:1000
GRID2	Abcam	ab198499	AB_2891030	1:5000
GRIK2	Abcam	ab124702	AB_10975460	1:1000
ARC	Abcam	ab183183	AB_2756512	1:1000
Anti-Rabbit IgG	Abcam	ab6721	AB_955447	1:5000

Abbreviations: ARC, activity-regulated cytoskeleton associated protein; GABRD, gamma-aminobutyric acid type A receptor subunit delta; GAPDH, glyceraldehyde-3-phosphate dehydrogenase; GRID2, glutamate ionotropic receptor delta type subunit 2; GRIK2, glutamate ionotropic receptor kainate type subunit 2.

### Statistical analysis

Statistical analysis was performed with R-package and GraphPad Prism software version 8.0. Group difference was analyzed by the Wilcoxon test and Mann–Whitney *U* test. According to the median value of SAPs, the samples in the data were divided into a low expression group and a high expression group. OS is presented as the Kaplan–Meier curve. Correlations were calculated using Pearson correlated coefficient. *P*<0.05 was regarded as statistically significant.

## Result

### Identification of differentially expressed synapse-associated proteins (SAPs) in lower-grade glioma (LGG)

**Figure 1:**
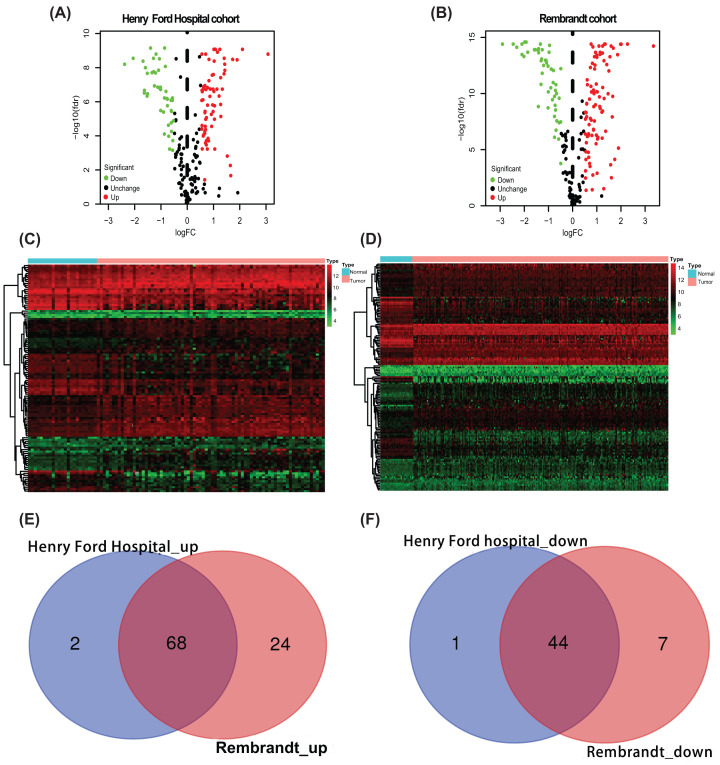
The differentially expressed SAPs between LGG and normal brain tissue Volcano plot showing the differentially expressed SAPs between LGG and normal brain tissues in the Henry Ford Hospital cohort (**A**) and Rembrandt cohort (**B**) Heatmap showing the differentially expressed SAPs between LGG and normal brain tissues in the Henry Ford Hospital cohort (**C**) and Rembrandt cohort (**D**) Venn showing common up-regulated SAPs (**E**) and down-regulated SAPs (**F**) in both cohorts; LGG, lower-grade glioma; SAP, synapse-associated protein.

The R software was adopted to identify the differentially expressed SAPs from a sum of 201 SAPs. A total of 112 SAPs was screened out for further analysis (*P*<0.05, | log2FC) | >0.5), which carried 44 down-regulated and 68 up-regulated SAPs (Figure 1, Table 3).

**Table 3:** Differentially expressed synapse-associated proteins (SAPs) in lower-grade gliomas

Category	genes	number
Up-regulated synapse-associated proteins	LRFN4, DHX36, CASK, EGLN1, PTN, FCGR2B, NTRK2, EIF4EBP1, ABHD17C, TTYH1, GRIA4, SSH1, IL1RAP, SRGN, ARC, CHRNA1, DBN1, SYT6, ACTL8, GRIK4, CEL, CACNG4, ARF4, CACNG6, ITSN1, FBXO45, ZNF804A, CAPRIN1, NLGN4X, LRP4, KPTN, SLC7A11, PTPRS, GRIK3, PDLIM5, NEDD4, PFN1, HOMER3, CACNG7, SEMA3F, CTTNBP2, LRRC4B, FYN, GHRL, NLGN2, POTEKP, GRIA3, NLGN1, EIF4A3, CRIPT, GRIK2, MAGI2, NLGN3, PLCB3, CYFIP1, HCLS1, ZMYND8, TANC2, DAG1, APOE, EPHB2, GRIK5, NLGN4Y, NRP2, FGF22, CRKL, CDH2, GRID2	68
Down-regulated synapse-associated proteins	PIN1, SYT5, STX1B, NGEF, HOMER1, GABRA1, NEFL, SYNPR, CACNB3, LZTS3, GABRA5, SRCIN1, SYN1, PPFIA2, DLG2, NRXN3, GRIN2B, GRIN2A, GABRA2, SV2A, CACNA1E, WASF1, SNAP91, NEFH, CACNA1B, CDK5, CDK5R1, EPHA4, NRN1, LRFN2, GRM5, CALB1, SYP, INA, SYN2, CACNG3, GABRB1, SNAP25, CAMKV, GABRD, PRKCG, CAMK2B, NCS1, ITPKA	44

### Functional enrichment analysis of the differently expressed SAPs

**Figure 2:**
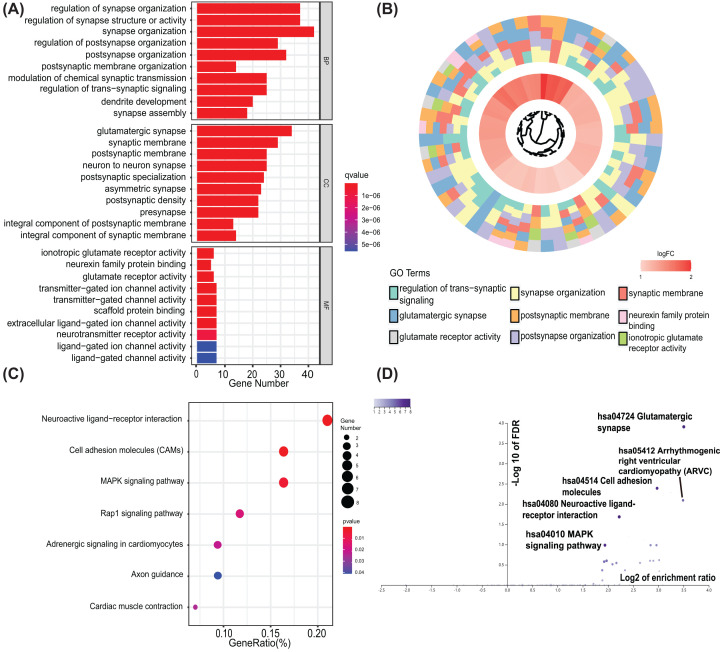
Function enrichment analysis on up-regulated SAPs (**A**) Bar plots respectively show the GO term up-regulated SAPs enriched in; (**B**) WEB-based Gene Set Analysis Toolkit was used to validate the result of GO analysis on up-regulated SAPs; (**C**) A bubble plot showing results of KEGG analyses for the up-regulated SAPs; (**D**) WEB-based Gene Set Analysis Toolkit was used to validate the result of and KEGG analysis on up-regulated SAPs; GO, gene ontology; KEGG, Kyoto Encyclopedia of Genes and Genomes; SAP, synapse-associated protein. GeneRatio, the ratio of the number of SAPs in the corresponding term to the total number of up-regulated SAPs.

**Figure 3:**
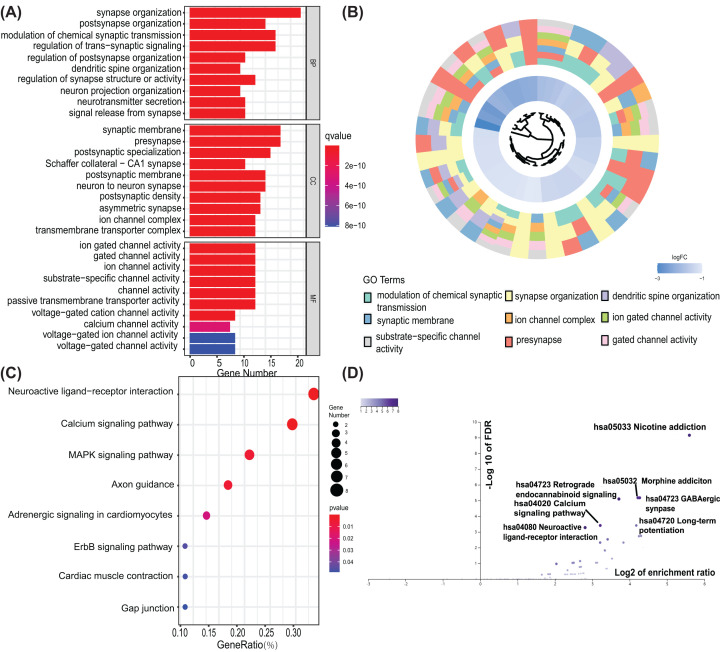
Function enrichment analysis on down-regulated SAPs (**A**) Bar plots respectively show the GO term down-regulated SAPs enriched in; (**B**) WEB-based Gene Set Analysis Toolkit was used to validate the result of GO analysis on down-regulated SAPs; (**C**) A bubble plot showing results of KEGG analyses for the down-regulated SAPs; (**D**) WEB-based Gene Set Analysis Toolkit was used to validate the result of and KEGG analysis on down-regulated SAPs; GO, gene ontology; KEGG, Kyoto Encyclopedia of Genes and Genomes; SAP, synapse-associated protein. GeneRatio, the ratio of the number of SAPs in the corresponding term to the total number of down-regulated SAPs.

To scrutinize the biofunction of identified SAPs, the SAP genes were grouped in accordance with their expression level. Subsequently, the online tool DAVID and WebGestalt was used to conduct a functional enrichment analysis of these groups. Gene otology (GO) indicated that the up-regulated differentially expressed SAPs were enriched in the synapse organization, glutamate receptor activity and postsynaptic membrane (Figure 2A,B). While the down-regulated differentially expressed SAPs were significantly enriched in modulation of chemical synaptic transmission, ion gated channel activity, gated channel activity, and presynapse (Figure 3A,B). Through Kyoto Encyclopedia of Genes and Genomes (KEGG) pathway analysis, we found that up-regulated SAPs were enriched in cell adhesion molecules pathway, glutamatergic synapse and neuroactive ligand–receptor interaction pathway (Figure 2C,D). Furthermore, down-regulated differentially expressed SAPs were mainly enriched in the calcium signaling and Nicotine addiction (Figure 3C,D).

### Construction of PPI network and key modules

**Figure 4:**
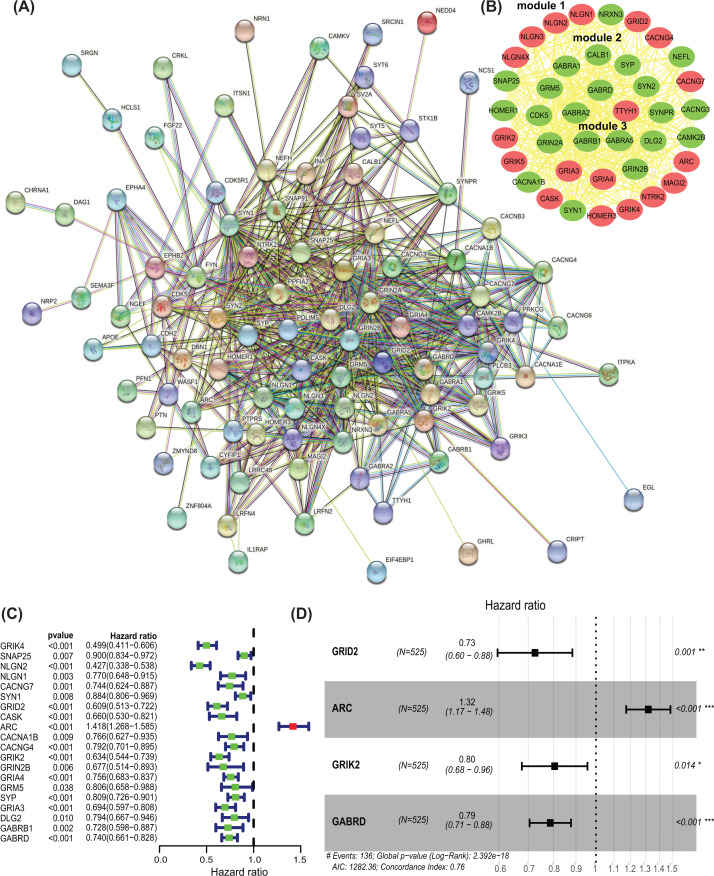
Identification of hub SAPs (**A**) Protein–protein interaction (PPI) network of differentially expressed SAPs; (**B**) key modules from the PPI network; (**C**) Univariate Cox regression analysis performed to identify prognosis-related SAPs in the training dataset; (**D**) Multivariate Cox regression analysis was performed to identify hub SAPs. Green circles: down-regulation; red circles: up-regulation; SAP, synapse-associated protein; *P<0.05; **P<0.01; ***P<0.001.

Based on the STRING database, the PPI network of differently expressed SAPs was constructed, which included 94 nodes and 563 edges (Figure 4A). Subsequently, the PPI network was analyzed to screen for potential three key modules using MODE in Cystoscope. Module 1 contained 23 nodes and 102 edges, module 2 included 12 nodes and 48 edges, module 3 consisted of 5 nodes and 10 edges (Figure 4B). Then, the function enrichment analysis demonstrated that the SAPs in module 1 were enriched in the glutamatergic synapse, glutamate receptor signaling pathway, synaptic membrane, neurexin family protein binding. The SAPs genes in module 2 were enriched in neuroactive ligand–receptor interaction, regulation of ion transmembrane transporter activity, synaptic membrane and glutamate receptor activity. The SAPs in module 3 were enriched in GABAergic synapse, chloride transmembrane transport, chloride channel complex and chloride channel activity.

### Selection of a prognostic-related SAPs

To analyze the prognostic significance of the key SAPs in key modules, univariate Cox regression analysis was used and obtained 20 prognostic-associated SAPs (Figure 4C). These candidate SAPs were analyzed by lasso regression analysis and multiple stepwise Cox regression analysis and four hub SAPs (ARC, GABRD, GRID2 and GRIK2) were identified as the independent predictors in LGG patients (Figure 4D and Supplementary Figure S1).

### Mutation and copy-number alteration analysis of hub SAPs in LGG

Mutation and copy-number alteration (CNA) analyses of SAPs were conducted using the GISTIC algorithm and segmentation analysis in cBioPortal6. The results showed that hub SAP genes were altered in 82 samples out of 2200 LGGs (4%) (Supplementary Figure S2A). However, alteration of these hub SAPs was not associated with the prognosis of LGGs (Supplementary Figure S2B and 2E).

### Validation of expression and prognostic value of hub SAPs in LGGs

**Figure 5:**
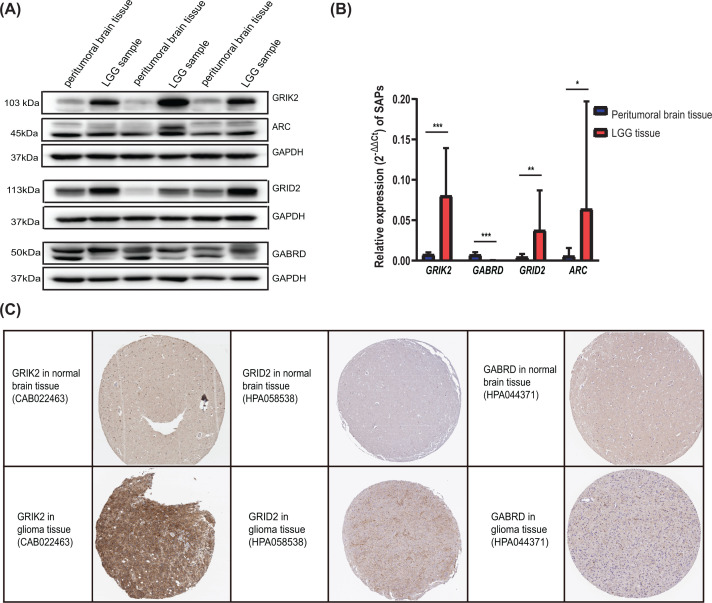
Hub SAPs expression and alteration analysis in LGGs Western blot (**A**) and qRT-PCR (**B**) were used to detect the transcript expression of hub SAPs between LGGs and peritumoral brain tissue. (**C**) Immunochemistry staining results from HPA database were used to detect the expression of hub SAPs; HPA, human protein atlas; SAP, synapse-associated protein; LGG, lower-grade glioma; *P<0.05; **P<0.01; ***P<0.001.

**Figure 6:**
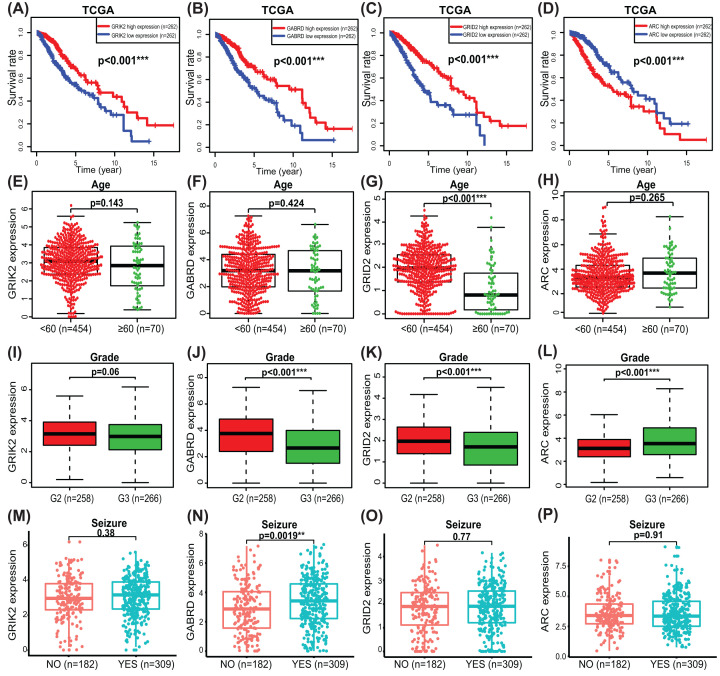
Prognostic value and expression characteristics of hub four SAPs in LGGs The prognostic value of GRIK2 (**A**); GABRD (**B**); GRID2 (**C**); ARC (**D**). The expression characteristics of hub SAPs in different age: GRIK2 (**E**); GABRD (**F**); GRID2 (**G**); ARC (**H**); The expression characteristics of hub SAPs in different grades: GRIK2 (**I**); GABRD (**J**); GRID2 (**K**); ARC (**L**); The expression characteristics of hub SAPs in group of seizure or not: GRIK2 (**M**); GABRD (**N**); GRID2 (**O**); ARC (**P**). ARC, activity-regulated cytoskeleton associated protein; GABRD, gamma-aminobutyric acid type A receptor subunit delta; GRID2, glutamate ionotropic receptor delta type subunit 2; GRIK2, glutamate ionotropic receptor kainate type subunit 2; LGG, lower-grade glioma; SAP, synapse-associated protein; *P<0.05; **P<0.01; ***P<0.001.

The expression level of the hub SAPs in LGG tissue and normal brain tissue was validated in Western blot and qRT-PCR (Figure 5A,B and Supplementary Figure S3). From the Human Protein Atlas database, immunohistochemistry results showed that GRID2 and GRIK2 were up-regulated in gliomas than normal brain tissue. On the contrary, GABRD were down-regulated in glioma tissue (Figure 5C). Furthermore, we used the Kaplan–Meier plotter method to probe the relationship between overall survival (OS) and expression of hub SAPs. Results indicated that the expression of hub SAPs was associated with the prognosis of LGG patients (Figure 6A–D). The expression of hub SAPs seems to correlate with LGG grades rather than age (Figure 6E–L). Besides, GABRD was up-regulated in LGG samples from patients with seizure than those without seizure (Figure 6N). The CGGA and Rembrandt cohorts were used to validate the above outcomes (Supplementary Figure S4).

**Figure 7:**
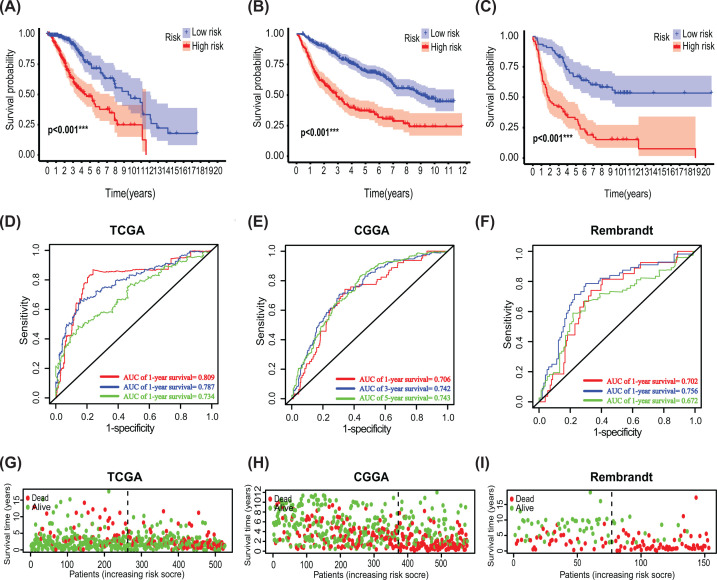
Analysis of the four-SAPs signature Survival analysis according to risk score in LGG of TCGA cohort (**A**), CGGA cohort (**B**) and Rembrandt cohort (**C**); ROC analysis on four-SAPs signature in TCGA cohort (**D**), CGGA cohort (**E**) and Rembrandt cohort (**F**); Survival status of patients in TCGA cohort (**G**), CGGA cohort (**H**) and Rembrandt cohort (**I**); CGGA, Chinese Glioma Genome Atlas; LGG, lower-grade glioma; ROC, receiver operating characteristic curve; SAP, synapse-associated protein; TCGA, The Cancer Genome Atlas; *P<0.05; **P<0.01; ***P<0.001.

### Construction and analysis of a four-SAPs signature

The predictive model was constructed on the basis of the expression of hub SAPs. The risk score of each patient was calculated according to the following formula:

$$\begin{array}{l} Risk\;score=(-0.19806*\text{Expression level of GRID2})+(0.248830*\text{Expression level of ARC})+\\ \left(-0.267494*\text{Expression level of GRIK2})+(-0.503747*\text{Expression level of GABRD}\right) \end{array}$$

Based on the median risk score, 524 LGG patients in the TCGA cohort were divided into low-risk and high-risk subgroups. Compared with patients in the low-risk subgroup, those in the high-risk subgroup exhibited a poorer OS (Figure 7A). Additionally, the prognostic value of the four-SAPs signature predictive model was assessed, and a similar formula was used to the CGGA and Rembrandt cohorts. Results revealed that LGG patients with high risk scores have a significantly lower OS than those with low risk scores (Figure 7B,C). Then, a time-dependent ROC analysis was performed, which suggested that it has a good diagnostic performance (Figure 7D–F). Besides, scatter plots were created to display the association between survival status of LGGs and the four-SAP signature (Figure 7G–I).

### Construction of nomogram based on the hub SAPs

**Figure 8:**
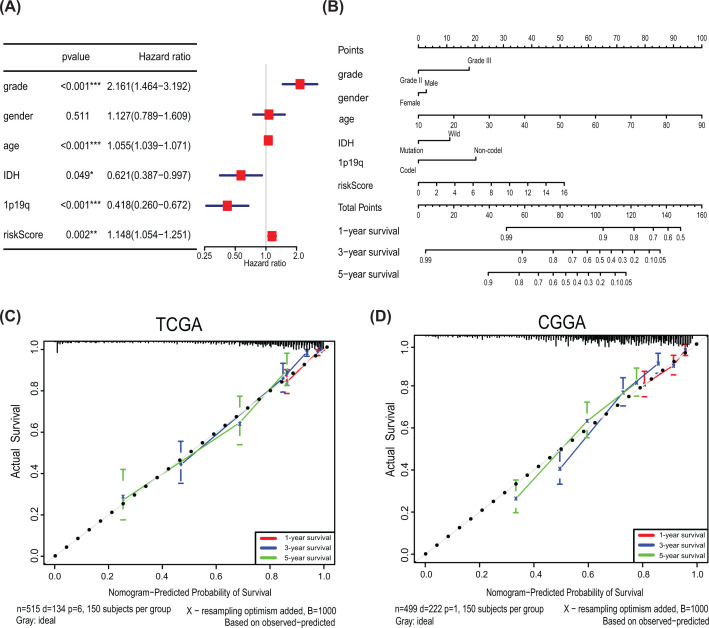
Nomogram and calibration plots of risk-signature and clinicopathologic factors (**A**) Based on multivariate Cox regression analysis, the risk score was an independent prognostic factor in LGGs. (**B**) Nomogram to predict 1-, 3- and 5-year OS in the TCGA cohort; Calibration plots of the nomogram to predict OS at 1, 3 and 5 years in the TCGA (**C**) and CGGA cohorts (**D**); CGGA, Chinese Glioma Genome Atlas; OS, overall survival; TCGA, The Cancer Genome Atlas; *P<0.05; **P<0.01; ***P<0.001.

Multivariate Cox regression analyses were used to estimate the prognostic significance of different clinical characteristics of LGG patients. Results indicated that the risk score was an independent prognostic factor associated with OS in glioma patients (*P*<0.05, Figure 8A). The nomogram and applied it in evaluating survival rates for LGG patients at 1, 3 and 5 years, which could aid clinicians in setting clinical plans for glioma patients (Figure 8B). The calibration plots presented good conformity between the predicted and observed outcomes (Figure 8C,D).

## Discussion

Non-specific treatments for glioma include surgical resection, radiotherapy and chemotherapy show a significant survival benefit in glioma patients [3]. However, there is still no targeted therapy effective against glioma. The abundance of neurotransmitters in the central nervous system creates a special microenvironment for brain tumors where cancer cells can transduce neurotransmitter-mediated intracellular signaling pathways inducing their growth, activation and metastasis [37]. Since neurotransmitters are important for tumor growth, the speculation that tumor cells might stimulate their innervation with neuron was proven by the discovery of neuro-glioma synapses and metastasis-neuron synapses [38]. These findings reveal a biologically crucial direct synaptic communication between neurons and tumor cells with potential clinical implications. However, as far as we know, the prognostic value of synapse-associated proteins in lower-grade glioma remains largely understudied.

In the present study, we performed functional enrichment analysis on differentially expressed SAPs. Biological processes or pathways that up-regulated SAPs are majorly enriched in synapse organization and special neurotransmitter receptor signals which include glutamate receptor signals. In the peripheral neural system, synapse modulation and neurotransmitter signals contribute to the progression of many tumors, including pancreatic cancer and prostate cancer [39,40]. In endometrial cancer (EC), GluR2 (GRIA2) expression was up-regulated, and the GluR2 antagonist effectively suppressed the invasion, migration and proliferation of tumor cells. Both *in vitro* and *in vivo*, EC cells showed its tropism toward DRG neurons and neuron fiber which implied that glutamate receptor signal and neuron–tumor interaction play a significant role in EC growth [41]. Moreover, in the central nervous system, previous studies demonstrated that the generation of synapse and glutamate receptor signals were linked to the progression of high-grade glioma and breast tumor patients, which corroborates with our results of LGG patients [16–18,42].

To further explore the association between SAPs in lower-grade gliomas (LGGs), a protein–protein interaction network of these differently expressed SAPs was created where three key modules including 40 key SAPs were screened. Among them, many SAPs have been reported to participate in the development and progression of tumors. Neuroligin-1 (NLGN1), neuroligin-2 (NLGN2), neuroligin-3 (NLGN3) and neuroligin-4X (NLGN4X) are neuroligin family members that interact with presynaptic neurexins to regulate heterophilic adhesion. In the deep brain region, a high expression of NLGN3 was associated with glioblastoma recurrence [43]. While in neuroblastoma, NLGN3 improved the phosphorylation level of Akt and up-regulated the transcription activity of the FOXO family hence promoting tumor proliferation [44]. In addition, Homer protein 3 (HOMER3), a member of HOMER, together with WW domain binding protein 2 (WBP2) plays a significant role in glioma invasion [45]. For instance, in leukemia, HOMER3 inhibits expression of Bcl2 thus influencing the cell cycle [46]. Activity-regulated cytoskeleton associated protein (ARC) causes the internalization of AMAPR from the postsynaptic membrane hence regulating the synapse strength. Through CaM kinase II modulatin, ARC promotes the neurite development in neuroblastoma [47,48]. As a multidomain scaffolding protein, calcium dependent serine protein kinase (CASK) interacts with several cytoplasmic adaptor proteins including Mint1, Reelin and NR2b by modulating synapse development and synaptic function [49–51]. Previous studies demonstrated that CASK is up-regulated in various tumors including, colorectal tumor, esophageal cancer, indicating that it might promote their progression or metastasis [52,53].

Through multivariate Cox regression models, four hub SAPs were screened out, including GRIK2 (glutamate ionotropic receptor kainate type subunit 2), GABRD (gamma-aminobutyric acid type A receptor subunit delta), GRID2 (glutamate ionotropic receptor delta type subunit 2) and ARC. Except for ARC, GRIK2 and GRID2, belonging to inotropic glutamatergic receptors, were shown to participate in neurodegeneration and psychiatric diseases, such as autism, Huntington disease and major depression [54,55]. There have been a few reports describing the relationship between these genes and cancers. A study by Zhang et al. demonstrated that the expression of GABRD might be negatively correlated with infiltrated macrophage in LGGs [56]. However, a better understanding of the molecular mechanisms of these four SAPs in glioma is paramount.

Several studies have discovered that seizure is the most prevalent symptom in glioma. Anti-epileptic drugs play a significant role in the combined treatment of glioma [57,58]. Glioma and glutamate disrupt the cortical networks and evoke peritumoral epileptic conditions [59,60]. Neuron-glioma synapse (NGS) plays the ‘bidirectional switch’ role. On the one hand, AMAP-induced current promotes post-synaptic glioma proliferation, and in turn, glioma increases neuronal activity through NGS, causing neuronal hyperexcitability and seizures [17,61,62]. Interestingly, our findings suggested that GABRD were up-regulated in the glioma tissue of patients with glioma-associated epilepsy compared with those in non-epileptic patients, indicating that SAPs may linked to the pathogenesis of seizures in glioma patients.

Last but not least, a risk model was constructed to predict the prognosis of LGG patients based on the hub SAPs. The ROC curve analysis confirmed that the risk model exhibited a better diagnostic capacity in identifying LGG patients with poor prognosis. Besides, a nomogram and were built to predict 3- and 5-year overall survival (OS). Using calibration plots, we evaluated the prediction capability of this clinical model in the CGGA cohort, whose results was consistent with that in the TCGA cohort.

## Conclusion

In the present study, we explored the prognostic value and expression of synapse-associated proteins LGG. Results revealed that the differentially expressed SAPs potentially participate in the formation of glutamate synapse, and synapse in LGG. Then, we constructed the prognostic model of four SAPs which acted as an independent prognostic factor in LGG. For the first time, our findings demonstrated that SAPs influence the pathogenesis of LGG, glioma-associated seizure and can potentially be novel prognostic molecular markers in glioma.

## Supplementary Material

Supplementary Figures S1-S4

Supplementary Table S1

## Data Availability

The datasets analyzed during the current study are available in the gene expression omnibus database (GEO, http://www.ncbi.nlm.nih.gov/geo), The Cancer Genome Atlas database (TCGA, https://portal.gdc.cancer.gov/) and Chinese Glioma Genome Atlas (CGGA, http://www.cgga.org.cn/). Immunohistochemistry staining results are available in The Human Protein Altas (HPA, https://www.proteinatlas.org/).

## Abbreviations

CGGA: Chinese Glioma Genome Atlas
DEG: differentially expressed gene
GEO: gene expression omnibus
HPA: human protein atlas
LGG: lower-grade glioma
OS: overall survival
SAP: synapse-associated protein
TCGA: The Cancer Genome Atlas
