# Supplementary material for: Validation of the functions and prognostic values of synapse-associated proteins in lower-grade glioma
Source: Biosci Rep. 2021 May 27;41(5):BSR20210391. doi: 10.1042/BSR20210391 (PMC8164110; doi:10.1042/BSR20210391)
Supplement: Supplementary Figures S1-S4 [file BSR-2021-0391_supp.pdf]

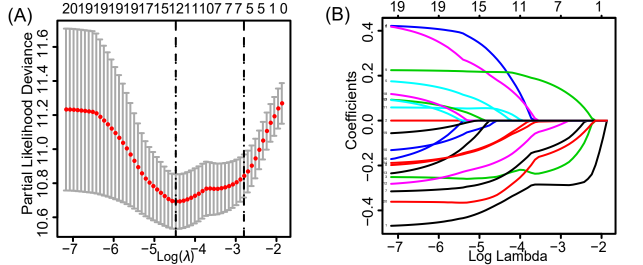

(A)
Partial Likelihood Deviance
Log(λ)
(B)
Coefficients
Log Lambda

Supplementary Figure 1. Lasso regression analysis of SAPs. (A) Partial Likelihood Deviance; (B) Coefficients of SAPs. SAP: synapse associated protein.

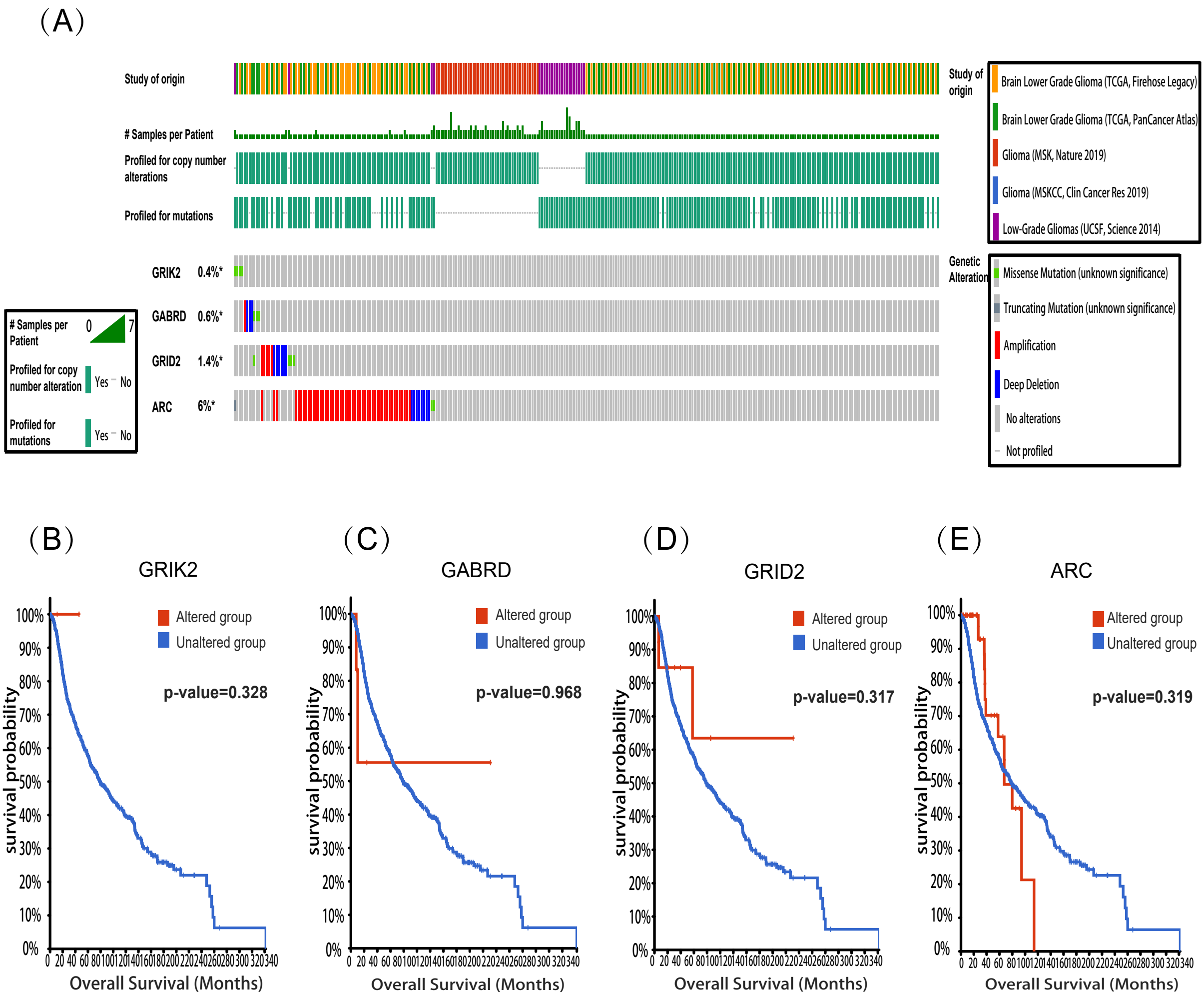
(A)
Study of origin
Samples per Patient
Profiled for copy number alterations
Profiled for mutations
GRIK2 0.4%*
GABRD 0.6%*
GRID2 1.4%*
ARC 6%*
Study of origin
Brain Lower Grade Glioma (TCGA, Firehose Legacy)
Brain Lower Grade Glioma (TCGA, PanCancer Atlas)
Glioma (MSK, Nature 2019)
Glioma (MSKCC, Clin Cancer Res 2019)
Low-Grade Gliomas (UCSF, Science 2014)
Genetic Alteration
Missense Mutation (unknown significance)
Truncating Mutation (unknown significance)
Amplification
Deep Deletion
No alterations
Not profiled
Samples per Patient 0 7
Profiled for copy number alteration Yes No
Profiled for mutations Yes No
(B)
GRIK2
Altered group
Unaltered group
p-value=0.328
(C)
GABRD
Altered group
Unaltered group
p-value=0.968
(D)
GRID2
Altered group
Unaltered group
p-value=0.317
(E)
ARC
Altered group
Unaltered group
p-value=0.319
survival probability
Overall Survival (Months)
100%
90%
80%
70%
60%
50%
40%
30%
20%
10%
0%
0 20 40 60 80 100 120 140 160 180 200 220 240 260 280 300 320 340

Supplementary Figure 2. Mutation and copy number variation of hub SAPs. (A) Copy-number alteration and mutation status of each hub gene; (B) the impact of alteration in GRIK2 status on the survival of LGGs; (C) the impact of alteration in GABRD status on the survival of LGGs; (D) the impact of alteration in GRID2 status on the survival of LGGs; (E) the impact of alteration in ARC status on the survival of LGGs. SAP: synapse associated protein; GRIK2: Glutamate Ionotropic Receptor Kainate Type Subunit 2; GABRD: Gamma-Aminobutyric Acid Type A Receptor Subunit Delta; GRID2: Glutamate Ionotropic Receptor Delta Type Subunit 2; ARC: Activity-regulated cytoskeleton associated protein.

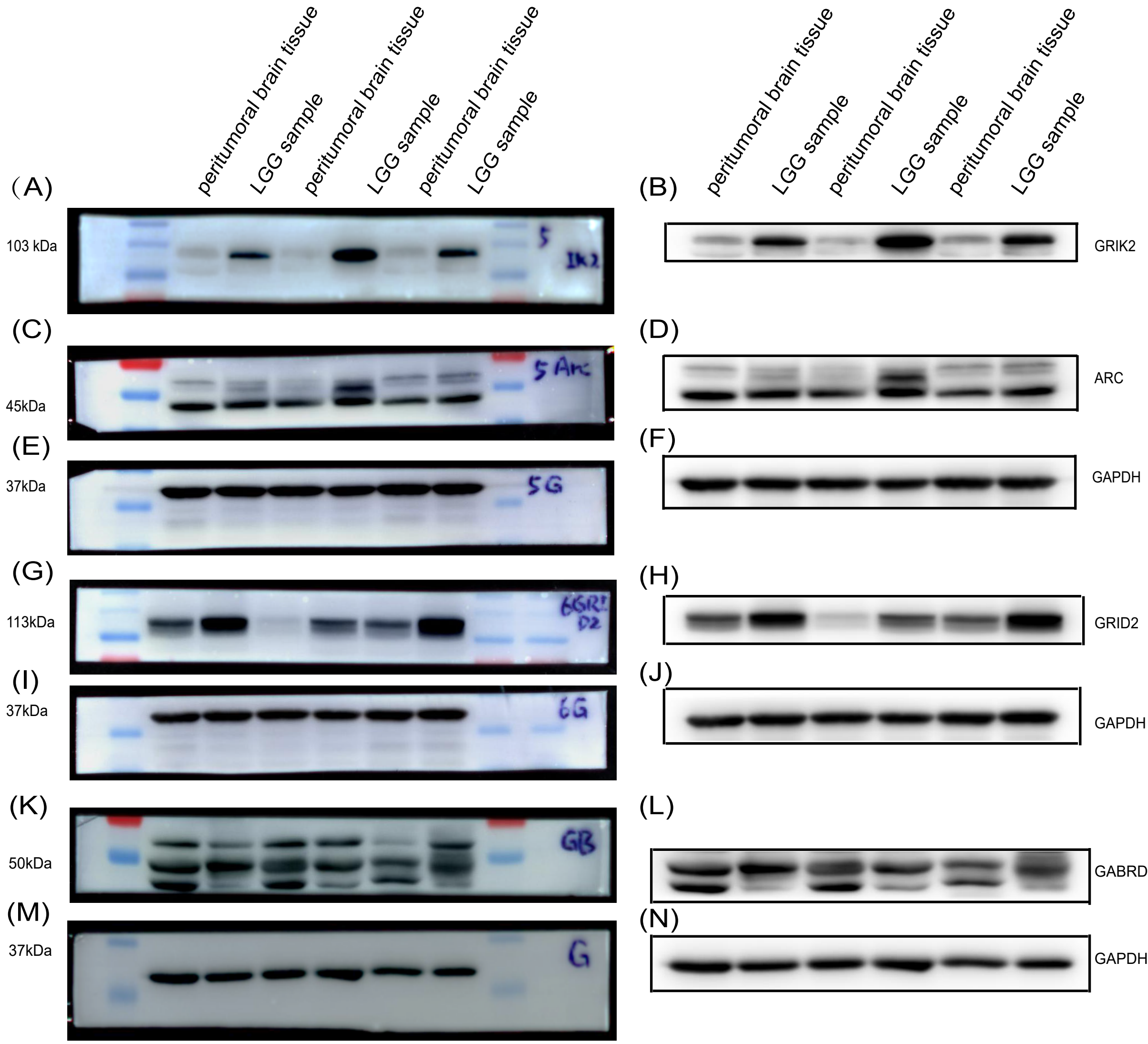
(A)
peritumoral brain tissue
LGG sample
peritumoral brain tissue
LGG sample
peritumoral brain tissue
LGG sample
103 kDa
(B)
GRIK2
(C)
45kDa
(D)
ARC
(E)
37kDa
(F)
GAPDH
(G)
113kDa
(H)
GRID2
(I)
37kDa
(J)
GAPDH
(K)
50kDa
(L)
GABRD
(M)
37kDa
(N)
GAPDH

Supplementary Figure 3. Western blot assay of the hub SAP expression in lower-grade glioma (LGG) samples and their corresponding peritumoral brain tissue. Original strip of GRIK2 (A) and corresponding outcome (B). Original strip of ARC (C) and corresponding outcome (D); Original strip of GAPDH (E) and corresponding outcome (F) together with the strips of GRIK2 and ARC; Original strip of GRID2 (G) and corresponding outcome (H); Original strip of GAPDH (I) and corresponding outcome (J) together with strip of GRID2; Original strip of GABRD (K) and corresponding outcome (L); Original strip of GAPDH (M) and corresponding outcome (N) together with strip of GABRD. PageRuler Prestained Protein Ladder (26616, Thermo, USA) was used to be molecular mass marker. SAP: synapse associated protein; LGG: lower-grade glioma; GRIK2: Glutamate Ionotropic Receptor Kainate Type Subunit 2; GABRD: Gamma-Aminobutyric Acid Type A Receptor Subunit Delta; GRID2: Glutamate Ionotropic Receptor Delta Type Subunit 2; ARC: Activity-regulated cytoskeleton associated protein. GAPDH: glyceraldehyde-3-phosphate dehydrogenase.

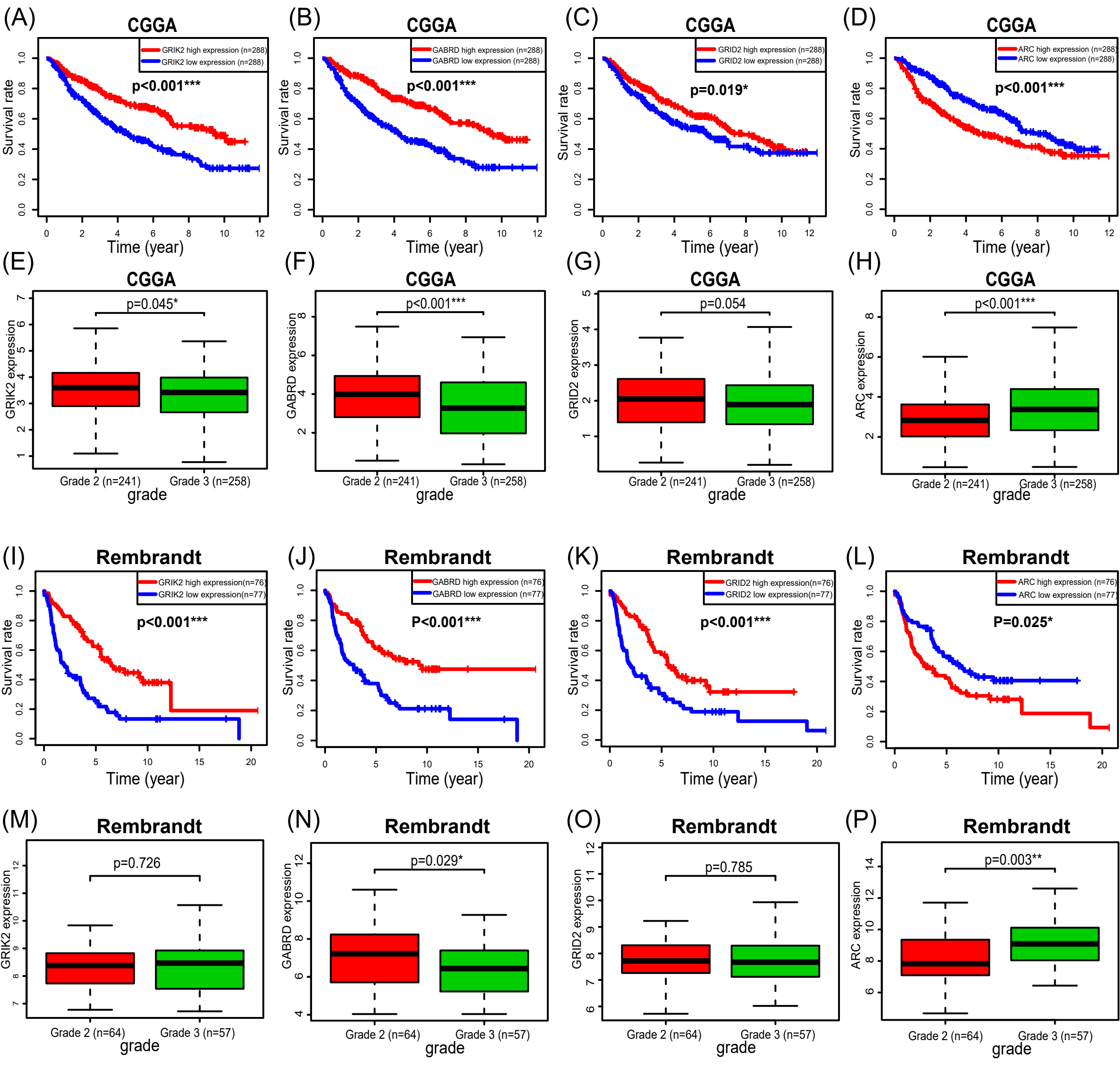

(A)
CGGA
GRIK2 high expression (n=288)
GRIK2 low expression (n=288)
p<0.001***
Survival rate
Time (year)
(B)
CGGA
GABRD high expression (n=288)
GABRD low expression (n=288)
p<0.001***
(C)
CGGA
GRID2 high expression (n=288)
GRID2 low expression (n=288)
p=0.019*
(D)
CGGA
ARC high expression (n=288)
ARC low expression (n=288)
p<0.001***
(E)
CGGA
p=0.045*
GRIK2 expression
Grade 2 (n=241)
Grade 3 (n=258)
grade
(F)
CGGA
p<0.001***
GABRD expression
(G)
CGGA
p=0.054
GRID2 expression
(H)
CGGA
p<0.001***
ARC expression
(I)
Rembrandt
GRIK2 high expression(n=76)
GRIK2 low expression(n=77)
p<0.001***
(J)
Rembrandt
GABRD high expression (n=76)
GABRD low expression (n=77)
P<0.001***
(K)
Rembrandt
GRID2 high expression(n=76)
GRID2 low expression(n=77)
p<0.001***
(L)
Rembrandt
ARC high expression (n=76)
ARC low expression (n=77)
P=0.025*
(M)
Rembrandt
p=0.726
Grade 2 (n=64)
Grade 3 (n=57)
(N)
Rembrandt
p=0.029*
(O)
Rembrandt
p=0.785
(P)
Rembrandt
p=0.003**

Supplementary Figure 4. CGGA and Rembrandt as invalidation cohorts. Overall survival of lower-grade glioma in CGGA cohort, including impact of GRIK2 (A), GABRD (B), GRID2 (C) and ARC (D) expression on overall survival in lower-grade glioma; In CGGA cohort, the expression characteristics of hub SAPs in different grades: GRIK2 (E); GABRD (F); GRID2 (G); ARC (H). Overall survival of lower-grade glioma in Rembrandt cohort, including impact of GRIK2 (I), GABRD (J), GRID2 (K) and ARC (L) expression on overall survival in lower-grade glioma; In Rembrandt cohort, the expression characteristics of hub SAPs in different grades: GRIK2 (M); GABRD (N); GRID2 (O); ARC (P). CGGA: Chinese Glioma Genome Atlas; GRIK2: Glutamate Ionotropic Receptor Kainate Type Subunit 2; GABRD: Gamma-Aminobutyric Acid Type A Receptor Subunit Delta; GRID2: Glutamate Ionotropic Receptor Delta Type Subunit 2; ARC: Activity-regulated cytoskeleton associated protein. *, $p<0.05$; **, $p<0.01$; ***, $p<0.001$.
